# Supplementary material for: On the mapping between evolutionary scenarios and governing rules in state-dependent speciation-extinction models
Source: bioRxiv. 2026 Sep 29:2026.09.24.754193. Preprint. [Version 1] doi: 10.64898/2026.09.24.754193 (PMC13648451; doi:10.64898/2026.09.24.754193)
Supplement: Supplementary file 1 [file NIHPP2026.09.24.754193v1-supplement-1.pdf]

bioRxiv preprint doi: https://doi.org/10.64898/2026.09.24.754193; this version posted September 29, 2026. The copyright holder for this preprint (which was not certified by peer review) is the author/funder, who has granted bioRxiv a license to display the preprint in perpetuity. It is made available under a CC-BY 4.0 International license.

# Supplementary Materials for: On the mapping between evolutionary scenarios and governing rules in state-dependent speciation-extinction models

## Contents

1.1 Proof of Theorem 1 . . . 2
1.2 Proof of Corollary 1 . . . 2
1.3 Proof of Corollary 2 . . . 4
1.4 Rulesets in a GeoSSE model . . . 5
1.5 Proof of Corollary S1 . . . 6
1.6 Proof of Lemma 1 . . . 7
1.7 Symmetries between rulesets in GeoSSE . . . 8
1.8 Proof of Corollary 3 . . . 10
1.9 Zero ruleset in GeoSSE . . . 10
1.10 Proof of Theorem 2 . . . 11
1.11 Proof of Lemma 2 . . . 13
1.12 Proof of Corollary 4 . . . 14
1.13 Number of non-trivial scenarios in BiSSE . . . 16
1.14 Number of non-trivial scenarios in 3-region GeoSSE . . . 17
1.15 Validation with randomized root states . . . 18
1.16 BiSSE simulation experiments . . . 19
1.17 Spectral gap analysis on simulated dataset . . . 20
1.18 Confusion matrix using trees with missing taxa and without rejection on imbalanced tip states . . . 21
1.19 Confusion matrix using trees with missing taxa and without rejection on imbalanced tip states and account for sampling proportion . . . 22
1.20 Confusion matrix using trees with complete sampling and with rejection on imbalanced tip states . . . 23
1.21 Confusion matrix using trees with missing taxa and with rejection on imbalanced tip states . . . 24
1.22 Confusion matrix using trees with missing taxa and with rejection on imbalanced tip states and account for sampling proportion . . . 25

bioRxiv preprint doi: https://doi.org/10.64898/2026.09.24.754193; this version posted September 29, 2026. The copyright holder for this preprint (which was not certified by peer review) is the author/funder, who has granted bioRxiv a license to display the preprint in perpetuity. It is made available under a CC-BY 4.0 International license.

## 1.1 Proof of Theorem 1

Let $N_i(t)$ denote the number of extant lineages in state $i$ at time $t$. Recall that $\hat{\mathbb{P}}_i^+$ and $\hat{\mathbb{P}}_i^-$ denote the probability of gaining or losing a new species in state $i$. At stationarity, the expected number of events that increase the number of lineages in state $i$ must balance the expected number of events that decrease it. Thus:

$$\hat{\mathbb{P}}_i^+ = \hat{\mathbb{P}}_i^-. \tag{1}$$

Next, because $\Pi_i(0)$ denotes the initial frequency of species in state $i$, the collection $\{\Pi_i(0)\}_{\forall i \in S}$ must form a valid probability distribution. Hence,

$$\sum_{i \in S} \Pi_i(0) = 1. \tag{2}$$

with $\Pi_i(0) \geq 0$, $\forall i \in S$. Likewise, the components of the speciation, extinction, and anagenetic transition-rate vectors represent event rates and are therefore restricted to the admissible positive parameter space.

To distinguish evolutionary rulesets associated with a given set of stationary frequencies, we additionally impose pairwise consistency between the ordering of the stationary frequencies and the corresponding total net rates. Thus, for every pair of states, $i, j \in S$, if $\hat{\Pi}_i = \hat{\Pi}_j$, we require the corresponding states are required to have equal total net rates, i.e. $\Phi_{total,i} = \Phi_{total,j}$. Similar requirements exist for the greater than and less than cases. Applying this condition to every pair $i, j \in S$ ensures that the ordering of the total net rates is consistent with the ordering of the given stationary frequencies and therefore defines the class of distinct evolutionary rulesets considered here. Hence, any parameter set satisfying the stationary balance equation and parameter-space constraints, together with the imposed pairwise ordering criterion, constitutes an admissible evolutionary ruleset associated with the given stationary frequencies. □

## 1.2 Proof of Corollary 1

Recall that $\hat{\mathbb{P}}_A^+$ and $\hat{\mathbb{P}}_A^-$ denote the probability of gaining or losing a new species in state $A$ under stationary scenario, respectively. In the BiSSE model, we can gain a new species in $A$ through a speciation event acting on a parent species in state $A$ and through an anagenetic change from state $B$ to state $A$. Therefore:

$$\hat{\mathbb{P}}_A^+ = \lambda_A \hat{\Pi}_A + q_{BA} \hat{\Pi}_B.$$

On the other hand, we lose a new species in $B$ through an extinction event for a species in state $B$ and through an anagenetic change from state $A$ to state $B$. Therefore:

$$\hat{\mathbb{P}}_A^- = (\mu_A + q_{AB}) \hat{\Pi}_A.$$

bioRxiv preprint doi: https://doi.org/10.64898/2026.09.24.754193; this version posted September 29, 2026. The copyright holder for this preprint (which was not certified by peer review) is the author/funder, who has granted bioRxiv a license to display the preprint in perpetuity. It is made available under a CC-BY 4.0 International license.

Similarly, we can define:

$$\hat{\mathbb{P}}_B^+ = \lambda_B \hat{\Pi}_B + q_{AB}\hat{\Pi}_A,$$

and

$$\hat{\mathbb{P}}_B^- = (\mu_B + q_{BA})\hat{\Pi}_B.$$

Assuming that there is no change in expected abundance of species in each state, we have:

$$\hat{\mathbb{P}}_A^+ = \hat{\mathbb{P}}_A^- \quad (3)$$
$$\hat{\mathbb{P}}_B^+ = \hat{\mathbb{P}}_B^- \quad (4)$$

$\Phi_{total,A}$ and $\Phi_{total,B}$ represent the flux between all incoming and outgoing rates from events occurring in state $A$ and $B$, respectively. In BiSSE, these come from the rates of speciation, extinction, and anagenetic change. Thus:

$$\begin{aligned}\Phi_{total,A} &= (\lambda_A + q_{BA}) - (\mu_A + q_{AB}) \\ &= (\lambda_A - \mu_A) + (q_{BA} - q_{AB}), \quad (5)\end{aligned}$$

and

$$\begin{aligned}\Phi_{total,B} &= (\lambda_B + q_{AB}) - (\mu_B + q_{BA}) \\ &= (\lambda_B - \mu_B) + (q_{AB} - q_{BA}). \quad (6)\end{aligned}$$

From Eqs (3) and (4), we have:

$$\begin{aligned}\lambda_A - \mu_A &= q_{AB} - q_{BA}\frac{\hat{\Pi}_B}{\hat{\Pi}_A}, \\ \lambda_B - \mu_B &= q_{BA} - q_{AB}\frac{\hat{\Pi}_A}{\hat{\Pi}_B}.\end{aligned}$$

Substituted into Eqs. (5) and (6) we have:

$$\begin{aligned}\Phi_{total,A} &= q_{AB} - q_{BA}\frac{\hat{\Pi}_B}{\hat{\Pi}_A} + q_{BA} - q_{AB} \quad (7) \\ &= q_{BA}\left(1 - \frac{\hat{\Pi}_B}{\hat{\Pi}_A}\right) \quad (8) \\ &= q_{BA}\left(\frac{\hat{\Pi}_A - \hat{\Pi}_B}{\hat{\Pi}_A}\right) \quad (9)\end{aligned}$$

bioRxiv preprint doi: https://doi.org/10.64898/2026.09.24.754193; this version posted September 29, 2026. The copyright holder for this preprint (which was not certified by peer review) is the author/funder, who has granted bioRxiv a license to display the preprint in perpetuity. It is made available under a CC-BY 4.0 International license.

$$\Phi_{total,B} = q_{BA} - q_{AB}\frac{\hat{\Pi}_A}{\hat{\Pi}_B} + q_{AB} - q_{BA} \tag{10}$$

$$= q_{AB}\left(1 - \frac{\hat{\Pi}_A}{\hat{\Pi}_B}\right) \tag{11}$$

$$= q_{AB}\left(\frac{\hat{\Pi}_B - \hat{\Pi}_A}{\hat{\Pi}_B}\right). \tag{12}$$

The relationship between $\Phi_{total,A}$ and $\Phi_{total,B}$ follows directly from the relationship between $\hat{\Pi}_A$ and $\hat{\Pi}_B$.

Furthermore, from Eqs. (3) and (4) we have:

$$(\lambda_A - \mu_A)\hat{\Pi}_A + (\lambda_B - \mu_B)\hat{\Pi}_B = 0.$$

For BiSSE models where there is no expected change in abundance of species in each state, when both stationary state frequencies are positive, $\hat{\Pi}_A, \hat{\Pi}_B > 0$, and both net diversification rates are nonzero, $\lambda_i \neq \mu_i, \forall i \in S$, the equation above requires the net diversification rates in states $A$ and $B$ to have opposite signs. Therefore, the net diversification rates of the two states cannot be equal. □

## 1.3 Proof of Corollary 2

The proof is similar to the proof of Corollary 1. However, here, instead of assuming zero net change in expected abundance in both states, we assume both states to grow at the equal rate $\rho$. Specifically,

$$\frac{dN_A(t)}{dt} = (\lambda_A - \mu_A - q_{AB})N_A + q_{BA}N_B \neq 0 \tag{13}$$

$$\frac{dN_A(t)}{dt} = (\lambda_B - \mu_B - q_{BA})N_B + q_{AB}N_A \neq 0. \tag{14}$$

Since $\hat{\Pi}_A$ and $\hat{\Pi}_B$ are stationary frequencies in $A$ and $B$, then the following is true as $t \to \infty$:

$$N_A(t) \approx \hat{\Pi}_A \times N(t) \tag{15}$$

$$N_B(t) \approx \hat{\Pi}_B \times N(t) \tag{16}$$

$$N(t) \propto \exp(\rho t). \tag{17}$$

Thus, using Eq. (17) and substituting both Eqs. (15) and (16) into Eqs. (13) and (14) after differentiating them w.r.t. $t$, we obtain:

$$\rho\hat{\Pi}_A \exp(\rho t) = (\lambda_A - \mu_A - q_{AB})\hat{\Pi}_A \exp(\rho t) + q_{BA}\hat{\Pi}_B \exp(\rho t) \tag{18}$$

$$\rho\hat{\Pi}_B \exp(\rho t) = (\lambda_B - \mu_B - q_{BA})\hat{\Pi}_B \exp(\rho t) + q_{AB}\hat{\Pi}_A \exp(\rho t), \tag{19}$$

bioRxiv preprint doi: https://doi.org/10.64898/2026.09.24.754193; this version posted September 29, 2026. The copyright holder for this preprint (which was not certified by peer review) is the author/funder, who has granted bioRxiv a license to display the preprint in perpetuity. It is made available under a CC-BY 4.0 International license.

which implies:

$$(\lambda_A - \mu_A - q_{AB})\hat{\Pi}_A + q_{BA}\hat{\Pi}_B = \rho \times \hat{\Pi}_A \tag{20}$$

$$(\lambda_B - \mu_B - q_{BA})\hat{\Pi}_B + q_{AB}\hat{\Pi}_A = \rho \times \hat{\Pi}_B. \tag{21}$$

Next, choosing $\rho > \max\left(q_{BA}\frac{\hat{\Pi}_B}{\hat{\Pi}_A} - q_{AB}, q_{AB}\frac{\hat{\Pi}_A}{\hat{\Pi}_B} - q_{BA}\right)$ ensures that $\lambda_A > \mu_A$ and $\lambda_B > \mu_B$ (i.e., both states are source). Similarly, choosing $\rho < \min\left(q_{BA}\frac{\hat{\Pi}_B}{\hat{\Pi}_A} - q_{AB}, q_{AB}\frac{\hat{\Pi}_A}{\hat{\Pi}_B} - q_{BA}\right)$ ensures that $\lambda_A < \mu_A$ and $\lambda_B < \mu_B$ (i.e., both states are sink). □

## 1.4 Rulesets in a GeoSSE model

We show how to derive a ruleset given a set of stationary frequencies of species count in different ranges in the context of a GeoSSE model (Goldberg et al. 2011). For simplicity, we describe in the case of $n = 2$ regions in Corollary S1.

**Corollary S1.**

*Given a GeoSSE with state space $S = \{A, B, AB\}$, set of stationary frequencies, $\left\{\hat{\Pi}_i, \forall i \in S\right\}$, and initial state frequencies $\{\Pi_A(0), \Pi_B(0), \Pi_{AB}(0) : \Pi_A(0) + \Pi_B(0) + \Pi_{AB}(0) = 1\}$. We obtain a ruleset(s) by solving the following system of equations:*

$$\begin{aligned}
&\hat{\mathbb{P}}^+_A = \hat{\mathbb{P}}^-_A \\
&\hat{\mathbb{P}}^+_B = \hat{\mathbb{P}}^-_B \\
&\hat{\mathbb{P}}^+_{AB} = \hat{\mathbb{P}}^-_{AB} \\
&\Phi_{total,i} \begin{cases} = \Phi_{total,j}, & \textit{if } \hat{\Pi}_i = \hat{\Pi}_j \\ > \Phi_{total,j}, & \textit{if } \hat{\Pi}_i > \hat{\Pi}_j \\ < \Phi_{total,j}, & \textit{if } \hat{\Pi}_i < \hat{\Pi}_j \end{cases} \\
&\sum_{i \in S} \Pi_i(0) = 1 \\
&\mathbf{\Psi}^s = \{w_A, w_B, b^A_B\} > \mathbf{0}, \mathbf{\Psi}^e = \{e_A, e_B\} > \mathbf{0}, \mathbf{\Psi}^a = \{d_{AB}, d_{BA}\} > \mathbf{0}, \Pi_i(0) \geq 0,\ \forall i, j \in S,
\end{aligned}$$

*where $w_A$ and $w_B$ are within-region speciation rates in region $A$ and $B$, respectively. $b^A_B$ is between-region speciation rates for widespread species $AB$ to speciate into two new species, each in region $A$ and $B$. $e_A$ and $e_B$ are local extinction rates in region $A$ and $B$. $d_{AB}$ and $d_{BA}$ are dispersal rates from region $A$ into region $B$ and from region $B$ into region $A$, respectively. For a 2-region GeoSSE model, we have:*

$$\begin{aligned}
\hat{\mathbb{P}}^+_A - \hat{\mathbb{P}}^-_A &= 0 \implies (w_A - d_{AB} - e_A)\hat{\Pi}_A + (w_A + b^A_B + e_B)\hat{\Pi}_{AB} = 0 \\
\hat{\mathbb{P}}^+_B - \hat{\mathbb{P}}^-_B &= 0 \implies (w_B - d_{BA} - e_B)\hat{\Pi}_B + (w_B + b^A_B + e_A)\hat{\Pi}_{AB} = 0 \\
\hat{\mathbb{P}}^+_{AB} - \hat{\mathbb{P}}^-_{AB} &= 0 \implies d_{AB}\hat{\Pi}_A + d_{BA}\hat{\Pi}_B - (e_A + e_B + b^A_B)\hat{\Pi}_{AB} = 0 \\
\Phi_{total,A} &= 2w_A + b^A_B + e_B - d_{AB} - e_A
\end{aligned}$$

bioRxiv preprint doi: https://doi.org/10.64898/2026.09.24.754193; this version posted September 29, 2026. The copyright holder for this preprint (which was not certified by peer review) is the author/funder, who has granted bioRxiv a license to display the preprint in perpetuity. It is made available under a CC-BY 4.0 International license.

$$\begin{aligned}\Phi_{total,B} &= 2w_B + b_B^A + e_A - d_{BA} - e_B \\ \Phi_{total,AB} &= d_{AB} + d_{BA} - e_A - e_B - b_B^A.\end{aligned}$$

**Proof:** Proof of Corollary S1 is given in Supplement 1.5. □

We demonstrate Corollary S1 in Example S2.

*Example S2* Suppose we have the following stationary frequencies under a 2-region GeoSSE model, $\hat{\Pi} = \{\frac{1}{3}, \frac{1}{3}, \frac{1}{3}\}$. Then, applying Corollary S1, we have two rulesets associated with the frequencies, given as follows:

**Ruleset 1**

$$\begin{aligned} w_A &= \frac{1}{2}\left(-2b_B^A + 2d_{AB} + d_{BA} - 2e_B\right), \\ w_B &= \frac{1}{2}\left(-d_{AB} + 2e_B\right), \\ e_A &= -b_B^A + d_{AB} + d_{BA} - e_B, \\ & b_B^A \le d_{AB} - e_B, \quad e_B < d_{AB} < 2e_B, \\ & w_A, w_B, e_A, e_B, d_{AB}, d_{BA}, b_B^A > 0. \end{aligned}$$

**Ruleset 2**

$$\begin{aligned} w_A &= \frac{1}{2}\left(-2b_B^A + 2d_{AB} + d_{BA} - 2e_B\right), \\ w_B &= \frac{1}{2}\left(-d_{AB} + 2e_B\right), \\ e_A &= -b_B^A + d_{AB} + d_{BA} - e_B, \\ & d_{AB} \le e_B, \quad d_{BA} > 2\left(b_B^A - d_{AB} + e_B\right), \\ & w_A, w_B, e_A, e_B, d_{AB}, d_{BA}, b_B^A > 0. \end{aligned}$$

For examples, the following parameters $w_A = 0.05, w_B = 0.05, e_A = 0.1, e_B = 0.2, d_{AB} = 0.3, d_{BA} = 0.1, b_B^A = 0.1$ satsify Ruleset 1 but does not satisfy Ruleset 2. On the other hand, $w_A = 0.05, w_B = 0.05, e_A = 0.3, e_B = 0.1, d_{AB} = 0.1, d_{BA} = 0.5, b_B^A = 0.2$ satisfy Ruleset 2 but does not satisfy Ruleset 1. Under both cases, they correspond to the same stationary frequencies $\hat{\Pi}$ using method given in Soewongsono and Landis (2024).

## 1.5 Proof of Corollary S1

In the context of a 2-region GeoSSE model, we can gain a new species with range state $A$ through 1) a within-region speciation event on endemic parent species in state $A$ or widespread parent species $AB$, 2) a between-region speciation event on widespread species $AB$, and 3) local extinction event in region $B$ by widespread species $AB$. Therefore:

$$\hat{\mathbb{P}}_A^+ = w_A \hat{\Pi}_A + \left(w_A + b_B^A + e_B\right)\hat{\Pi}_{AB}.$$

On the other hand, we can lose a new species in $A$ through 1) a local extinction event in region $A$ and 2) anagenetic change through dispersal event to a widespread species

bioRxiv preprint doi: https://doi.org/10.64898/2026.09.24.754193; this version posted September 29, 2026. The copyright holder for this preprint (which was not certified by peer review) is the author/funder, who has granted bioRxiv a license to display the preprint in perpetuity. It is made available under a CC-BY 4.0 International license.

$AB$. Therefore:

$$\hat{\mathbb{P}}_A^- = (d_{AB} + e_A)\hat{\Pi}_A.$$

Similarly, we can define:

$$\hat{\mathbb{P}}_B^+ = w_B\hat{\Pi}_B + \left(w_B + b_B^A + e_A\right)\hat{\Pi}_{AB},$$

and

$$\hat{\mathbb{P}}_B^- = (d_{BA} + e_B)\hat{\Pi}_B.$$

Next, we can gain a widespread species $AB$ through a dispersal event from species in region $A$ to region $B$, and vice versa. We can lose a widespread species through a local extinction event in region $A$ and region $B$, and through a between-region speciation event. Therefore:

$$\hat{\mathbb{P}}_{AB}^+ = d_{AB}\hat{\Pi}_A + d_{BA}\hat{\Pi}_B,$$

and

$$\hat{\mathbb{P}}_{AB}^- = \left(e_A + e_B + b_B^A\right)\hat{\Pi}_{AB}.$$

$\Phi_{total,A}$ and $\Phi_{total,B}$ represent the flux between all incoming and outgoing rates from events occurring in state $A$ and $B$, respectively. In a GeoSSE model, the incoming rates are from within-region speciation on both endemic and widespread species and from between-region speciation and local extinction on widespread species. While, the outgoing rates are from local extinction event on endemic species. Thus:

$$\Phi_{total,A} = \left(2w_A + b_B^A + e_B\right) - (d_{AB} + e_A),$$

and

$$\Phi_{total,B} = \left(2w_B + b_B^A + e_A\right) - (d_{BA} + e_B),$$

As for the widespread species, we have

$$\Phi_{total,AB} = (d_{AB} + d_{BA}) - \left(e_A + e_B + b_B^A\right).$$

□

## 1.6 Proof of Lemma 1

The proof follows directly from Theorem 1. Suppose we have an arbitrary pair of symmetric state frequencies namely, $\hat{\mathbf{\Pi}}_1$ and $\hat{\mathbf{\Pi}}_2$. Suppose the rate parameters that correspond to $\hat{\mathbf{\Pi}}_2$ are obtained by re-labelling the indices in rate parameters that

bioRxiv preprint doi: https://doi.org/10.64898/2026.09.24.754193; this version posted September 29, 2026. The copyright holder for this preprint (which was not certified by peer review) is the author/funder, who has granted bioRxiv a license to display the preprint in perpetuity. It is made available under a CC-BY 4.0 International license.

correspond to $\hat{\mathbf{\Pi}}_1$. Then, in both patterns, we solve the same system of equations and constraints as described in Theorem 1. Thus, they both return the same solution sets. In other words, both pairs of symmetric state patterns return symmetric rulesets after re-labelling the indices on rate parameters.

□

## 1.7 Symmetries between rulesets in GeoSSE

*Example S3* Suppose a 2-region GeoSSE model with state space $S = \{A, B, AB\}$ with $\mathbf{\Psi}^s = \{w_A, w_B, b_B^A\} > \mathbf{0}, \mathbf{\Psi}^e = \{e_A, e_B\} > \mathbf{0}, \mathbf{\Psi}^a = \{d_{AB}, d_{BA}\} > \mathbf{0}$. Then, given a pair of symmetric state frequencies as follows:

$$\hat{\mathbf{\Pi}}_1 = \{0.1, 0.2, 0.7\},\ \hat{\mathbf{\Pi}}_2 = \{0.2, 0.1, 0.7\}. \tag{22}$$

Then, using Corollary S1 (solved using `Mathematica` (Wolfram Research, Inc. 2023)), rulesets that correspond to $\hat{\mathbf{\Pi}}_1$ are as follows:

1. **Ruleset 1**

$$w_A = \frac{1}{8}\left(-7b_B^A + d_{AB} + \frac{1}{7}(-7b_B^A + d_{AB} + 2d_{BA} - 7e_B) - 7e_B\right)$$
$$w_B = \frac{1}{9}(-d_{AB} + 9e_B)$$
$$e_A = \frac{1}{7}(-7b_B^A + d_{AB} + 2d_{BA} - 7e_B)$$
$$0 < b_B^A < \frac{1}{28}(4d_{AB} + d_{BA} - 28e_B),\ 0 < d_{BA} < \frac{14d_{AB}}{9},\ 7e_B \leq d_{AB} < 9e_B,\ e_B > 0.$$

2. **Ruleset 2**

$$w_A = \frac{1}{8}\left(-7b_B^A + d_{AB} + \frac{1}{7}(-7b_B^A + d_{AB} + 2d_{BA} - 7e_B) - 7e_B\right)$$
$$w_B = \frac{1}{9}(-d_{AB} + 9e_B)$$
$$e_A = \frac{1}{7}(-7b_B^A + d_{AB} + 2d_{BA} - 7e_B)$$
$$0 < b_B^A < \frac{1}{28}(4d_{AB} + d_{BA} - 28e_B),\ \frac{126e_B}{25} < d_{AB} < 7e_B,\ -4d_{AB} + 28e_B < d_{BA} < \frac{14d_{AB}}{9},\ e_B > 0.$$

On the other hand, rulesets that correspond to $\hat{\mathbf{\Pi}}_2$ are as follows:

1. **Ruleset 1**

$$w_A = \frac{1}{9}\left(-7b_B^A + 2d_{AB} + \frac{2}{7}\left(-7b_B^A + 2d_{AB} + d_{BA} - 7e_B\right) - 7e_B\right)$$
$$w_B = \frac{1}{8}(-2d_{AB} + 8e_B)$$
$$e_A = \frac{1}{7}\left(-7b_B^A + 2d_{AB} + d_{BA} - 7e_B\right)$$

bioRxiv preprint doi: https://doi.org/10.64898/2026.09.24.754193; this version posted September 29, 2026. The copyright holder for this preprint (which was not certified by peer review) is the author/funder, who has granted bioRxiv a license to display the preprint in perpetuity. It is made available under a CC-BY 4.0 International license.

$$0 < b_B^A < \frac{1}{63}(18d_{AB} + 2d_{BA} - 63e_B),\ 0 < d_{AB} \leq \frac{49e_B}{15},\ d_{BA} > \frac{1}{2}(-18d_{AB} + 63e_B),\ e_B > 0.$$

2. **Ruleset 2**

$$w_A = \frac{1}{9}\left(-7b_B^A + 2d_{AB} + \frac{2}{7}\left(-7b_B^A + 2d_{AB} + d_{BA} - 7e_B\right) - 7e_B\right)$$
$$w_B = \frac{1}{8}(-2d_{AB} + 8e_B)$$
$$e_A = \frac{1}{7}\left(-7b_B^A + 2d_{AB} + d_{BA} - 7e_B\right)$$
$$0 < b_B^A < \frac{1}{63}(18d_{AB} + 2d_{BA} - 63e_B),\ \frac{49e_B}{15} < d_{AB} < 4e_B,\ d_{BA} > \frac{9d_{AB}}{14},\ e_B > 0.$$

Note that `Mathematica` has a tendency to preserve the order of how expressions are being represented, depending on user's order of input parameters. This leads to the symmetry between rulesets for a pair of symmetrical stationary state frequencies is being **indirectly** represented. However, the ruleset 1 for $\hat{\mathbf{\Pi}}_1$ is indeed symmetric to the ruleset 2 for $\hat{\mathbf{\Pi}}_2$. Similarly, ruleset 2 for $\hat{\mathbf{\Pi}}_1$ is symmetric to the ruleset 1 for $\hat{\mathbf{\Pi}}_2$. To show the symmetries, we can re-write both rulesets in $\hat{\mathbf{\Pi}}_2$ (equivalent to changing the order of input parameters in `Mathematica`) as follows:

1. **Ruleset 1**

$$w_A = \frac{1}{9}(-d_{BA} + 9e_A)$$
$$w_B = \frac{1}{8}\left(-7b_B^A + d_{BA} + \frac{1}{7}\left(-7b_B^A + 2d_{AB} + d_{BA} - 7e_A\right) - 7e_A\right)$$
$$e_B = \frac{1}{7}\left(-7b_B^A + 2d_{AB} + d_{BA} - 7e_A\right)$$
$$0 < b_B^A < \frac{1}{28}(4d_{BA} + d_{AB} - 28e_A),\ \frac{126e_A}{25} < d_{BA} < 7e_A,\ -4d_{BA} + 28e_A < d_{AB} < \frac{14d_{BA}}{9},\ e_A > 0.$$

2. **Ruleset 2**

$$w_A = \frac{1}{9}(-d_{BA} + 9e_A)$$
$$w_B = \frac{1}{8}\left(-7b_B^A + d_{BA} + \frac{1}{7}\left(-7b_B^A + 2d_{AB} + d_{BA} - 7e_A\right) - 7e_A\right)$$
$$e_B = \frac{1}{7}\left(-7b_B^A + 2d_{AB} + d_{BA} - 7e_A\right)$$
$$0 < b_B^A < \frac{1}{28}(4d_{BA} + d_{AB} - 28e_A),\ 0 < d_{AB} < \frac{14d_{BA}}{9},\ 7e_A \leq dba < 9e_A,\ e_A > 0. \quad \square$$

bioRxiv preprint doi: https://doi.org/10.64898/2026.09.24.754193; this version posted September 29, 2026. The copyright holder for this preprint (which was not certified by peer review) is the author/funder, who has granted bioRxiv a license to display the preprint in perpetuity. It is made available under a CC-BY 4.0 International license.

## 1.8 Proof of Corollary 3

From Corollary 1 we have,

$$\begin{aligned}
\hat{\mathbb{P}}_A^+ - \hat{\mathbb{P}}_A^- &= 0 \implies (\lambda_A - \mu_A - q_{AB})\hat{\Pi}_A + q_{BA}\hat{\Pi}_B = 0 && (23)\\
\hat{\mathbb{P}}_B^+ - \hat{\mathbb{P}}_B^- &= 0 \implies (\lambda_B - \mu_B - q_{BA})\hat{\Pi}_B + q_{AB}\hat{\Pi}_A = 0. && (24)
\end{aligned}$$

**Case 1:** $\hat{\Pi}_A = 0$

Then, from Eq (23) we have:

$$q_{BA}\hat{\Pi}_B = 0. \tag{25}$$

However, since $\hat{\Pi}_B = 1 - \hat{\Pi}_A$, from Eq. (25) we have $\hat{\Pi}_A = 1$ since $q_{BA} \neq 0$. This is a contradiction.

**Case 2:** $\hat{\Pi}_B = 0$

Proof for Case 2 follows directly from proof for Case 1 by re-labelling the rate parameters. By symmetry, $\hat{\Pi}_B = 1$ since $q_{AB} \neq 0$. This is a contradiction. □

## 1.9 Zero ruleset in GeoSSE

**Corollary S4.** *Suppose we have a 2-region GeoSSE model with state space $S = \{A, B, AB\}$ with rate parameters $w_A, w_B, e_A, e_B, d_{AB}, d_{BA}, b_B^A > 0$. Given a set of stationary frequencies $\{\hat{\Pi}_A, \hat{\Pi}_B, \hat{\Pi}_{AB}\}$, there is no non-trivial ruleset satisfying the system of equations in Corollary S1 if either*
*(i) at least one stationary frequency is zero, or*
*(ii) $\hat{\Pi}_{AB}$ is equal to exactly one of $\hat{\Pi}_A$ or $\hat{\Pi}_B$.*

*Proof* We first show for case (i). From Corollary S1 we have:

$$\begin{aligned}
\hat{\mathbb{P}}_A^+ - \hat{\mathbb{P}}_A^- &= 0 \implies \hat{\Pi}_A(w_A - d_{AB} - e_A) + \hat{\Pi}_{AB}(w_A + b_B^A + e_B) = 0 && (26)\\
\hat{\mathbb{P}}_B^+ - \hat{\mathbb{P}}_B^- &= 0 \implies \hat{\Pi}_B(w_B - d_{BA} - e_B) + \hat{\Pi}_{AB}(w_B + b_B^A + e_A) = 0 && (27)\\
\hat{\mathbb{P}}_{AB}^+ - \hat{\mathbb{P}}_{AB}^- &= 0 \implies \hat{\Pi}_A(d_{AB}) + \hat{\Pi}_B(d_{BA}) - \hat{\Pi}_{AB}(e_A + e_B + b_B^A) = 0. && (28)
\end{aligned}$$

**Scenario 1:** $\hat{\Pi}_A = 0$

Then, from Eq. (26) we have:

$$\hat{\Pi}_{AB}(w_A + b_B^A + e_B) = 0.$$

Since $\hat{\Pi}_{AB} \neq 0$, this implies that $w_A + b_B^A + e_B = 0$. However, by assumption, $w_A, b_B^A, e_B > 0$. Thus, it is a contradiction.

**Scenario 2:** $\hat{\Pi}_B = 0$

bioRxiv preprint doi: https://doi.org/10.64898/2026.09.24.754193; this version posted September 29, 2026. The copyright holder for this preprint (which was not certified by peer review) is the author/funder, who has granted bioRxiv a license to display the preprint in perpetuity. It is made available under a CC-BY 4.0 International license.

From Eq. (27) we have

$$\hat{\Pi}_{AB}(w_B + b_B^A + e_A) = 0$$

Since $\hat{\Pi}_{AB} \neq 0$, this implies that $w_B + b_B^A + e_A = 0$. However, by assumption, $w_B, b_B^A, e_A > 0$. Thus, it is a contradiction.

**Scenario 3:** $\hat{\Pi}_{AB} = 0$

From Eq. (28) we have:

$$\hat{\Pi}_A(d_{AB}) + \hat{\Pi}_B(d_{BA}) = 0.$$

By assumption, $d_{AB}, d_{BA} > 0$. Thus, $\hat{\Pi}_A = \hat{\Pi}_B = 0$. However, this is a contradiction. Next, to show case (ii), we assume that $\hat{\Pi}_B = \hat{\Pi}_{AB}$, and all stationary frequencies are positive. The proof for $\hat{\Pi}_A = \hat{\Pi}_{AB}$ follows by symmetry. From Eq. (27) we have

$$2w_B + b_B^A e_A - d_{BA} - e_B = 0$$

since $\hat{\Pi}_B = \hat{\Pi}_{AB} > 0$. By definition, it implies

$$\Phi_{total,B} = 0.$$

Moreover, $\Phi_{total,AB} = 0$ since $\hat{\Pi}_B = \hat{\Pi}_{AB}$. Then, we have

$$d_{AB} + d_{BA} - e_A - e_B - b_B^A = 0$$
$$\therefore d_{AB} - e_A - e_B - b_B^A = -d_{BA}.$$

From Eq. (28) we have

$$d_{AB}\left(\hat{\Pi}_A - \hat{\Pi}_B\right) = 0. \tag{29}$$

This implies $d_{AB} = 0$ since $\hat{\Pi}_A \neq \hat{\Pi}_B$. This is a contradiction since all rates are positive. □

## 1.10 Proof of Theorem 2

First, we prove the equalities that define $\lambda_A$ and $\lambda_B$ in rulesets $1a/b$ and $2a/b$. By re-arranging parameters in Eqs. (4) and (5) in Corollary 1, we have:

$$\lambda_A = \mu_A + q_{AB} - \frac{\hat{\Pi}_B}{\hat{\Pi}_A} q_{BA}, \tag{30}$$

$$\lambda_B = \mu_B + q_{BA} - \frac{\hat{\Pi}_A}{\hat{\Pi}_B} q_{AB}. \tag{31}$$

Then, the proof for $\lambda_A$ and $\lambda_B$ in rulesets $1c$ and $2c$ follows directly since $\hat{\Pi}_A = \hat{\Pi}_B$.

Next, we consider case $\hat{\Pi}_A < \hat{\Pi}_B$ to prove the inequalities in ruleset $1a$. Let's suppose we have rate corresponds to species gain in state $A$. That is, $\lambda_A > \mu_A$. Since $\lambda_A > \mu_A$ we have:

$$\mu_A + q_{AB} - \frac{\hat{\Pi}_B}{\hat{\Pi}_A} q_{BA} > \mu_A \tag{32}$$

$$\therefore 0 < q_{BA} < \frac{\hat{\Pi}_A}{\hat{\Pi}_B} q_{AB}, \text{ since } q_{BA} > 0 \text{ by assumption.} \tag{33}$$

bioRxiv preprint doi: https://doi.org/10.64898/2026.09.24.754193; this version posted September 29, 2026. The copyright holder for this preprint (which was not certified by peer review) is the author/funder, who has granted bioRxiv a license to display the preprint in perpetuity. It is made available under a CC-BY 4.0 International license.

Next, by assumption $\lambda_B > 0$ we have:

$$\mu_B + q_{BA} - \frac{\hat{\Pi}_A}{\hat{\Pi}_B} q_{AB} > 0, \tag{34}$$

$$\therefore \mu_B > \frac{\hat{\Pi}_A q_{AB} - \hat{\Pi}_B q_{BA}}{\hat{\Pi}_B}. \tag{35}$$

It also follows that $\lambda_B < \mu_B$ by Eqs. (31) and (33). This completes the proof for ruleset $1a$. Next, assuming we have rate corresponds to species gain in $B$. That is $\lambda_B > \mu_B$. Since $\lambda_B > \mu_B$ we have

$$\mu_B + q_{BA} - \frac{\hat{\Pi}_A}{\hat{\Pi}_B} q_{AB} > \mu_B, \tag{36}$$

$$\therefore q_{BA} > \frac{\hat{\Pi}_A}{\hat{\Pi}_B} q_{AB}. \tag{37}$$

Next, by assumption $\lambda_A$ we have:

$$\mu_A + q_{AB} - \frac{\hat{\Pi}_B}{\hat{\Pi}_A} q_{BA} > 0, \tag{38}$$

$$\therefore \mu_A > \frac{\hat{\Pi}_B q_{BA} - \hat{\Pi}_A q_{AB}}{\hat{\Pi}_A}. \tag{39}$$

It also follows that $\lambda_A < \mu_A$ by Eqs. (30) and (37). This completes the proof for ruleset $2a$. Next, for case $\hat{\Pi}_A > \hat{\Pi}_B$, the proof for the inequalities in rulesets $1b$ and $2b$ follow directly by swapping the state indices in the inequalities for rulesets $1a$ and $2a$, respectively. Next, we consider case $\hat{\Pi}_A = \hat{\Pi}_B$ to prove the inequalities in rulesets $1c$ and $2c$. Consider $\lambda_B > \mu_B$. It follows from Eq. (37) that:

$$q_{BA} > q_{AB}. \tag{40}$$

Next, from Eq. (39) we have:

$$\mu_A > q_{BA} - q_{AB}. \tag{41}$$

This completes the proof for ruleset $1c$. Next, assuming $\lambda_A > \mu_A$, it follows from Eq. (33) that:

$$q_{BA} < q_{AB}. \tag{42}$$

Next, from Eq. (35) we have:

$$\mu_B > q_{AB} - q_{BA}. \tag{43}$$

This completes the proof for ruleset $2c$. □

bioRxiv preprint doi: https://doi.org/10.64898/2026.09.24.754193; this version posted September 29, 2026. The copyright holder for this preprint (which was not certified by peer review) is the author/funder, who has granted bioRxiv a license to display the preprint in perpetuity. It is made available under a CC-BY 4.0 International license.

### 1.11 Proof of Lemma 2

Following the proof in Theorem 2 for ruleset 1$a$, we have $\lambda_A > \mu_A$ and $\lambda_B < \mu_B$. Moreover, we have:

$$q_{BA} < \frac{\hat{\Pi}_A}{\hat{\Pi}_B} q_{AB}. \tag{44}$$

Since ruleset 1$a$ corresponds to $\hat{\Pi}_A < \hat{\Pi}_B$, it follows that $q_{BA} < q_{AB}$. This completes the proof of the mapping between ruleset 1$a$ and scenario 1$a$.

Next, following the proof in Theorem 2 for ruleset 2$a$, we have $\lambda_A < \mu_A$ and $\lambda_B > \mu_B$. Moreover, Eq. (37) still holds regardless of whether $q_{AB} < q_{BA}$ or $q_{AB} > q_{BA}$. This completes the proof of the mapping between ruleset 2$a$ and scenario 2$a$. Next, by symmetry, the proof for scenarios 1$b$ and 2$b$ follows directly from 1$a$ and 2$a$.

Following the proof in Theorem 2 for ruleset 1$c$, we have $\lambda_B > \mu_B$. Since ruleset 1$c$ corresponds to $\hat{\Pi}_A = \hat{\Pi}_B$, it follows from Eq. (30) that:

$$\lambda_A = \mu_A - (q_{BA} - q_{AB}), \tag{45}$$

and so, $\lambda_A < \mu_A$ since $q_{BA} > q_{AB}$. This completes the proof of the mapping between ruleset 1$c$ and scenario 1$c$. Next, following the proof in Theorem 2 for ruleset 2$c$, we have $\lambda_A > \mu_A$. Moreover, since $q_{BA} < q_{AB}$ it follows from Eq. (31) that $\lambda_B < \mu_B$. This completes the proof of the mapping between ruleset 2$c$ and scenario 2$c$.

Finally, in order to show the other non-trivial scenarios described by the Lemma, it is sufficient to show their existence and their inherent exclusion by the method described in Corollary 1. Let's assume they are included by the method.

In scenario 3$a$ and 4$a$, we have $\lambda_A > \mu_A$ and $\lambda_B > \mu_B$. Therefore, it follows from Eqs. (30) and (31) that:

$$q_{BA} > \frac{\hat{\Pi}_A}{\hat{\Pi}_B} q_{AB}, \text{ and} \tag{46}$$

$$q_{BA} < \frac{\hat{\Pi}_A}{\hat{\Pi}_B} q_{AB}. \tag{47}$$

However, this is a contradiction. Hence, using proof-by-contradiction, scenarios 3$a$ and 4$a$ are excluded by the method and so, we cannot derive explicit rulesets for these scenarios using the method described in Corollary 1. The existence of scenarios 3$a$ and 4$a$ follows directly from Corollary 2. That is, we have $\lambda_A > \mu_A$ and $\lambda_B > \mu_B$. Furthermore, $q_{BA}\frac{\hat{\Pi}_B}{\hat{\Pi}_A} - q_{AB} > q_{AB}\frac{\hat{\Pi}_A}{\hat{\Pi}_B} - q_{BA}$ implies $q_{BA} > q_{AB}$, and $q_{BA}\frac{\hat{\Pi}_B}{\hat{\Pi}_A} - q_{AB} < q_{AB}\frac{\hat{\Pi}_A}{\hat{\Pi}_B} - q_{BA}$ implies $q_{BA} < q_{AB}$. This completes the proof for scenarios 3$a$ and 4$a$. By symmetry, the proof for scenarios 3$b$ and 4$b$ also follows in a similar manner.

Next, let's assume we can derive scenarios 5$a$ and 6$a$ using method described in Corollary 1. Then, given that $\lambda_A < \mu_A$ and $\lambda_B < \mu_B$, it follows from Eqs. (30) and

bioRxiv preprint doi: https://doi.org/10.64898/2026.09.24.754193; this version posted September 29, 2026. The copyright holder for this preprint (which was not certified by peer review) is the author/funder, who has granted bioRxiv a license to display the preprint in perpetuity. It is made available under a CC-BY 4.0 International license.

(31) that:

$$q_{BA} < \frac{\hat{\Pi}_A}{\hat{\Pi}_B} q_{AB}, \text{ and} \tag{48}$$

$$q_{BA} > \frac{\hat{\Pi}_A}{\hat{\Pi}_B} q_{AB}. \tag{49}$$

However, this is a contradiction. The existence of scenarios 5$a$ and 6$b$ follows directly from Corollary 2. That is, we have $\lambda_A < \mu_A$ and $\lambda_B < \mu_B$. Furthermore, $q_{BA}\frac{\hat{\Pi}_B}{\hat{\Pi}_A} - q_{AB} > q_{AB}\frac{\hat{\Pi}_A}{\hat{\Pi}_B} - q_{BA}$ implies $q_{BA} > q_{AB}$, and $q_{BA}\frac{\hat{\Pi}_B}{\hat{\Pi}_A} - q_{AB} < q_{AB}\frac{\hat{\Pi}_A}{\hat{\Pi}_B} - q_{BA}$ implies $q_{BA} < q_{AB}$. This completes the proof for scenarios 5$a$ and 6$a$. By symmetry, the proof for scenarios 5$b$ and 6$b$ also follows in a similar manner.

Next, in Scenario 7$a$ we have $\lambda_A - \mu_A = \lambda_B - \mu_B$. Then, using Eqs. (30) and (31) we have:

$$q_{AB} - \frac{\hat{\Pi}_B}{\hat{\Pi}_A} q_{BA} = q_{BA} - \frac{\hat{\Pi}_A}{\hat{\Pi}_B} q_{AB}. \tag{50}$$

$$\therefore q_{AB} = \frac{\hat{\Pi}_B}{\hat{\Pi}_A} q_{BA}. \tag{51}$$

Substitute Eq. (51) to Eq. (4) in Corollary 1 we have:

$$(\lambda_A - \mu_A)\hat{\Pi}_A - 0. \tag{52}$$

However, since $\hat{\Pi}_A \neq 0$. Then, it implies $\lambda_A = \mu_A$, which is a contradiction. However, this scenario does exist. If the rate of species accumulation in both state through speciation are equal, then the direction of the transition rate determines the direction of the stationary class. In this case, since $q_{AB} > q_{BA}$, then it follows that $\hat{\Pi}_A < \hat{\Pi}_B$. This completes the proof for scenario 7$a$. By symmetry, the proof for scenario 7$b$ follows in a similar manner.

For scenario 3$c$, as usual, first we show that there is no ruleset that corresponds to this scenario under the method described in Corollary 1. Then, we show the existence of such scenario. Since $q_{AB} = q_{BA}$, it follows from Eq. (4) in Corollary 1 that $\lambda_A = \mu_A$, which is a contradiction. Next, we show the existence of the scenario. By the assumption on the rate parameters under this scenario, it follows that $\hat{\Pi}_A = \hat{\Pi}_B$ since it is equivalent to a state-independent diversification model. □

## 1.12 Proof of Corollary 4

*Proof* From Corollary 2 we have:

$$M\hat{\mathbf{\Pi}} = \rho\hat{\mathbf{\Pi}}, \tag{53}$$

where

$$M = \begin{pmatrix} \lambda_A - \mu_A - q_{AB} & q_{BA} \\ q_{AB} & \lambda_B - \mu_B - q_{BA} \end{pmatrix}; \hat{\mathbf{\Pi}} = \begin{pmatrix} \hat{\Pi}_A \\ \hat{\Pi}_B \end{pmatrix}. \tag{54}$$

bioRxiv preprint doi: https://doi.org/10.64898/2026.09.24.754193; this version posted September 29, 2026. The copyright holder for this preprint (which was not certified by peer review) is the author/funder, who has granted bioRxiv a license to display the preprint in perpetuity. It is made available under a CC-BY 4.0 International license.

Eq. (53) defines that, at equilibrium, the net contribution to each state is proportional to its abundance and each state grows at the same rate $\rho$. Therefore, the resulting state frequencies are constant over time. Using quadratic formula, the eigenvalues of $M$ are given by:

$$\rho_{1,2} = \frac{a + b \pm \sqrt{(a+b)^2 - 4(ab - q_{AB}q_{BA})}}{2} \tag{55}$$

$$= \frac{a + b \pm \sqrt{(a-b)^2 + 4q_{AB}q_{BA}}}{2}. \tag{56}$$

where

$$a = \lambda_A - \mu_A - q_{AB}, \tag{57}$$

$$b = \lambda_B - \mu_B - q_{BA}. \tag{58}$$

Note that discriminant in Eq. (56) is positive, therefore both eigenvalues are distinct and real. Denote the dominant eigenvalue $\rho = \rho_1$. It follows that the spectral gap is given by:

$$\gamma = \sqrt{(a-b)^2 + 4q_{AB}q_{BA}} \tag{59}$$

$$= \sqrt{[(r_A - r_B) + q_{BA} - q_{AB}]^2 + 4q_{AB}q_{BA}}, \tag{60}$$

where $r_A = \lambda_A - \mu_A$ and $r_B = \lambda_B - \mu_B$ are net diversification rates. By definition, it follows that the relaxation time is given by:

$$\hat{t} = \frac{1}{\gamma}. \tag{61}$$

□

bioRxiv preprint doi: https://doi.org/10.64898/2026.09.24.754193; this version posted September 29, 2026. The copyright holder for this preprint (which was not certified by peer review) is the author/funder, who has granted bioRxiv a license to display the preprint in perpetuity. It is made available under a CC-BY 4.0 International license.

297 ## 1.13 Number of non-trivial scenarios in BiSSE

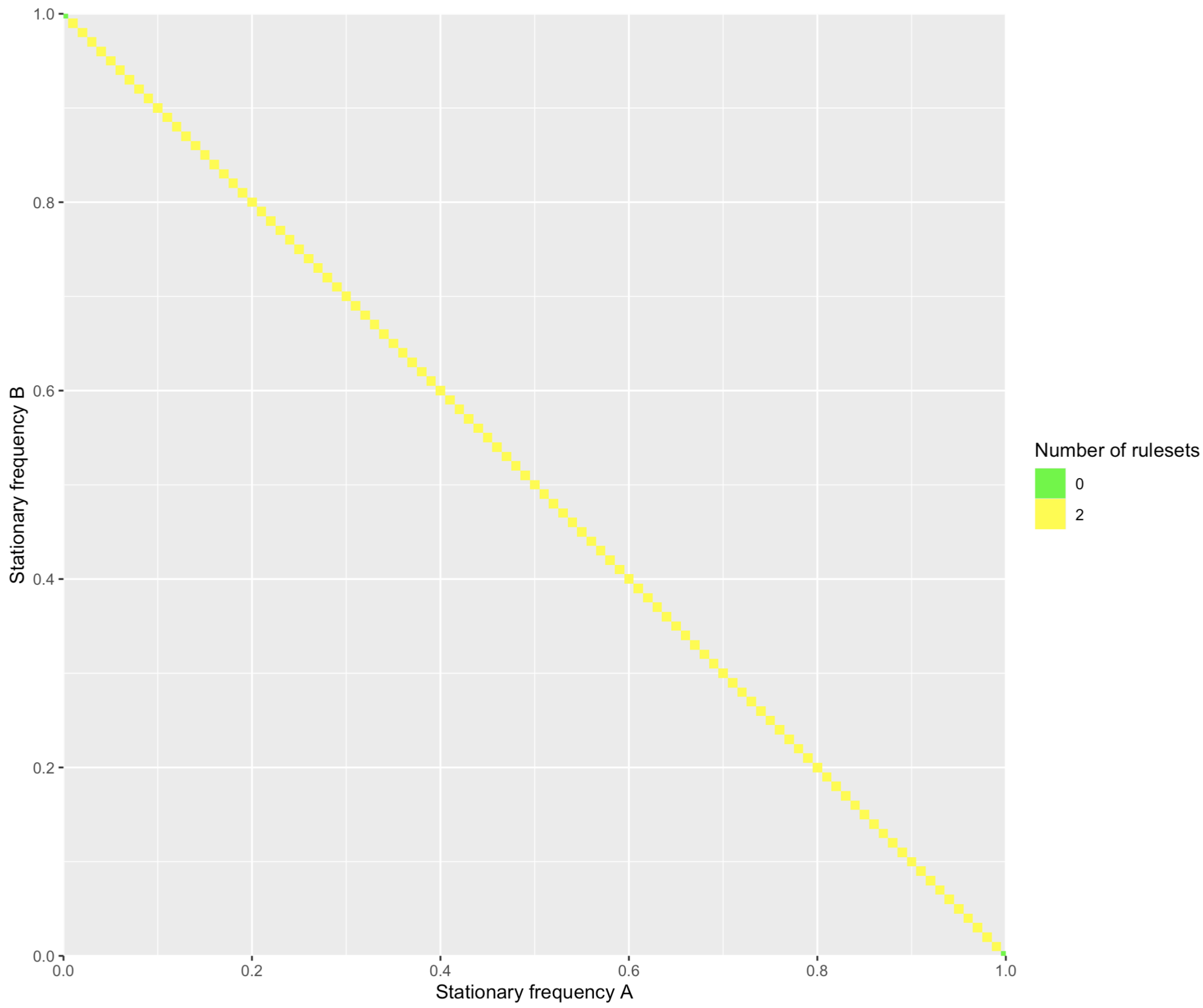


**Fig. S1**: Number of non-trivial rulesets associated with each set of stationary frequencies sampled from a hypercube under the BiSSE model. Because $\hat{\Pi}_A + \hat{\Pi}_B = 1$, all possible stationary frequency combinations lie along the diagonal. Two non-trivial rulesets exist for $0 < \hat{\Pi}_A, \hat{\Pi}_B < 1$, whereas no ruleset exists at the boundary cases $\hat{\Pi}_A = 0$ or $\hat{\Pi}_B = 0$ under the assumption that all model rates are positive.

bioRxiv preprint doi: https://doi.org/10.64898/2026.09.24.754193; this version posted September 29, 2026. The copyright holder for this preprint (which was not certified by peer review) is the author/funder, who has granted bioRxiv a license to display the preprint in perpetuity. It is made available under a CC-BY 4.0 International license.

298 ## 1.14 Number of non-trivial scenarios in 3-region GeoSSE

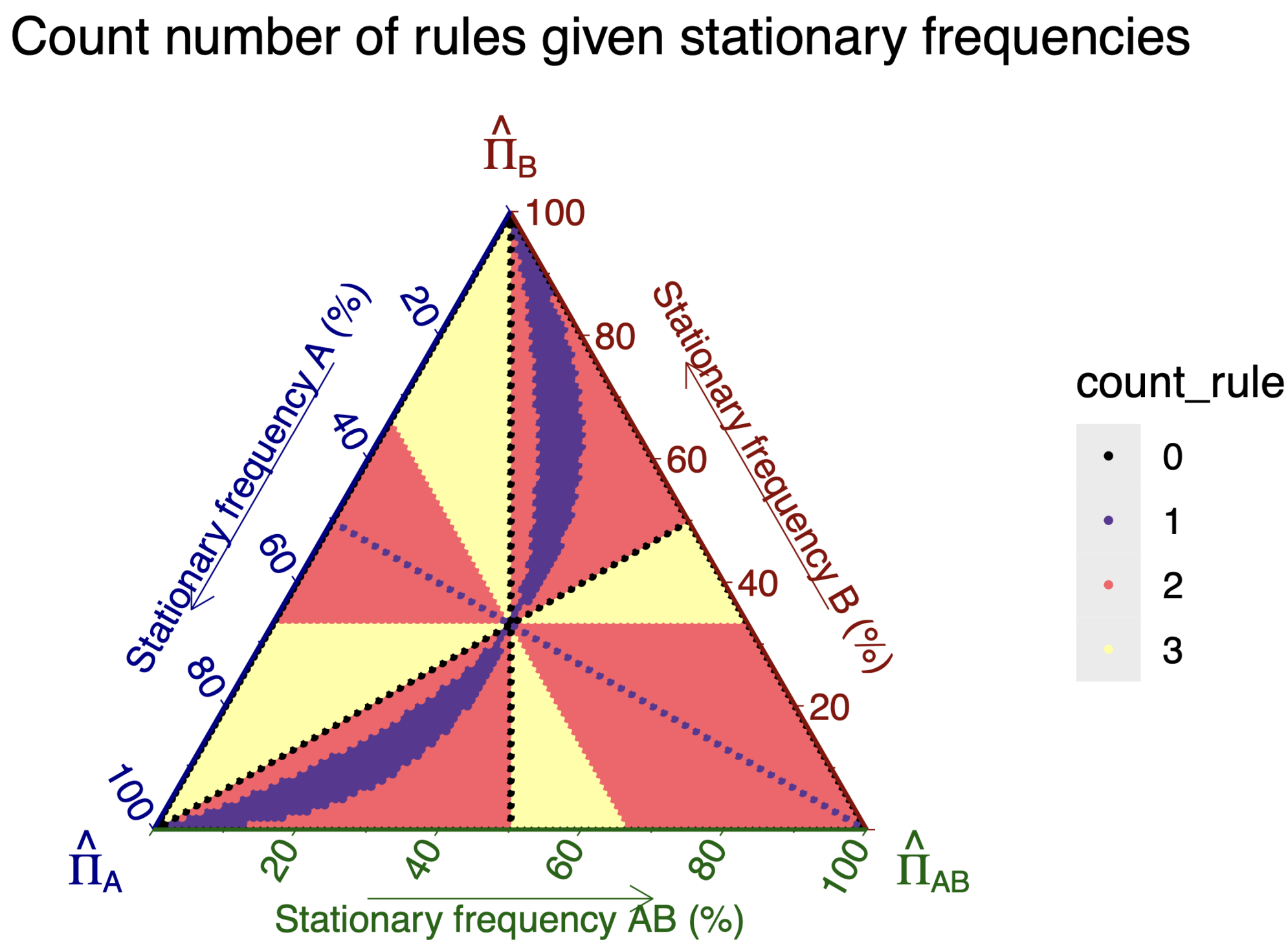


**Fig. S2**: Ternary plot showing the number of non-trivial rulesets associated with each set of stationary frequencies sampled from a hypercube under the 2-region GeoSSE model.

bioRxiv preprint doi: https://doi.org/10.64898/2026.09.24.754193; this version posted September 29, 2026. The copyright holder for this preprint (which was not certified by peer review) is the author/funder, who has granted bioRxiv a license to display the preprint in perpetuity. It is made available under a CC-BY 4.0 International license.

## 1.15 Validation with randomized root states

**Table S1**: Distribution of true evolutionary scenarios among valid trees, each with 100 extant taxa, under the randomized root validation experiment. The table reports the number of trees, with the relative prevalence in parentheses, for each true scenario among all valid trees. The initial state was randomly assigned to state $A$ or $B$ with equal probability. Trees were simulated under the complete-noreject setting without rejection based on tip-state frequencies.

| **Experiment** | **Evolutionary scenario** | | | | | | | | | | | | | | | | |
|---|---|---|---|---|---|---|---|---|---|---|---|---|---|---|---|---|---|
| | 1a | 2a | 3a | 4a | 5a | 6a | 7a | 1b | 2b | 3b | 4b | 5b | 6b | 7b | 1c | 2c | 3c |
| $\mathbb{P}(S_j \mid \text{valid})$ | 26 (1.30%) | 415 (20.75%) | 531 (26.55%) | 67 (3.35%) | – | – | – | 36 (1.80%) | 362 (18.10%) | 492 (24.60%) | 71 (3.55%) | – | – | – | – | – | – |

bioRxiv preprint doi: https://doi.org/10.64898/2026.09.24.754193; this version posted September 29, 2026. The copyright holder for this preprint (which was not certified by peer review) is the author/funder, who has granted bioRxiv a license to display the preprint in perpetuity. It is made available under a CC-BY 4.0 International license.

300 ## 1.16 BiSSE simulation experiments

**Table S2**: Distribution of the true parameter relationships for simulated trees classified into evolutionary scenarios 1a−4a. The single value reports the median, whereas the interval reports the first and third quartiles of each parameter ratio.

| Experiment | 1a | | 2a | | 3a | | 4a | |
|---|---|---|---|---|---|---|---|---|
| | $\frac{\lambda_B-\mu_B}{\lambda_A-\mu_A}$ | $\frac{q_{AB}}{q_{BA}}$ | $\frac{\lambda_B-\mu_B}{\lambda_A-\mu_A}$ | $\frac{q_{AB}}{q_{BA}}$ | $\frac{\lambda_B-\mu_B}{\lambda_A-\mu_A}$ | $\frac{q_{AB}}{q_{BA}}$ | $\frac{\lambda_B-\mu_B}{\lambda_A-\mu_A}$ | $\frac{q_{AB}}{q_{BA}}$ |
| complete-noreject | −0.24<br>[−0.39, −0.10] | 2.44<br>[1.62, 3.31] | −3.07<br>[−6.56, −1.43] | 1.54<br>[0.96, 2.84] | 1.11<br>[0.55, 2.07] | 2.11<br>[1.53, 3.31] | 2.22<br>[1.75, 5.08] | 0.84<br>[0.67, 0.95] |
| missing-noreject<br>& missing-noreject-sampling | −0.16<br>[−0.28, −0.05] | 2.50<br>[1.97, 3.77] | −2.73<br>[−5.51, −1.42] | 1.64<br>[1.04, 2.69] | 1.21<br>[0.65, 2.11] | 2.02<br>[1.46, 3.08] | 3.13<br>[2.22, 11.04] | 0.81<br>[0.67, 0.92] |
| complete-reject | −0.13<br>[−0.29, −0.06] | 1.97<br>[1.61, 2.72] | −4.65<br>[−8.57, −1.91] | 1.06<br>[0.91, 1.31] | 1.00<br>[0.51, 1.63] | 1.52<br>[1.24, 1.96] | 3.05<br>[2.12, 6.85] | 0.85<br>[0.77, 0.93] |
| missing-reject<br>& missing-reject-sampling | −0.18<br>[−0.31, −0.07] | 2.25<br>[1.87, 3.60] | −4.00<br>[−6.23, −2.00] | 1.02<br>[0.85, 1.21] | 0.96<br>[0.46, 1.63] | 1.51<br>[1.21, 2.00] | 3.15<br>[2.00, 9.68] | 0.85<br>[0.77, 0.94] |

**Table S3**: Distribution of the true parameter relationships for simulated trees classified into evolutionary scenarios 1b–4b. The single value reports the median, whereas the interval reports the first and third quartiles of each parameter ratio.

| Experiment | 1b | | 2b | | 3b | | 4b | |
|---|---|---|---|---|---|---|---|---|
| | $\frac{\lambda_A-\mu_A}{\lambda_B-\mu_B}$ | $\frac{q_{BA}}{q_{AB}}$ | $\frac{\lambda_A-\mu_A}{\lambda_B-\mu_B}$ | $\frac{q_{BA}}{q_{AB}}$ | $\frac{\lambda_A-\mu_A}{\lambda_B-\mu_B}$ | $\frac{q_{BA}}{q_{AB}}$ | $\frac{\lambda_A-\mu_A}{\lambda_B-\mu_B}$ | $\frac{q_{BA}}{q_{AB}}$ |
| complete-noreject | −0.08<br>[−0.38, −0.04] | 3.08<br>[2.37, 5.60] | −2.33<br>[−4.74, −1.36] | 1.53<br>[1.04, 2.83] | 1.33<br>[0.83, 2.50] | 2.00<br>[1.35, 3.28] | 5.27<br>[2.56, 19.49] | 0.79<br>[0.68, 0.89] |
| missing-noreject<br>& missing-noreject-sampling | −0.05<br>[−0.13, −0.02] | 3.21<br>[1.97, 5.60] | −2.11<br>[−4.34, −1.37] | 1.61<br>[0.98, 2.71] | 1.64<br>[0.89, 3.82] | 2.21<br>[1.43, 3.37] | 5.27<br>[2.38, 12.04] | 0.79<br>[0.67, 0.88] |
| complete-reject | −0.10<br>[−0.29, −0.05] | 2.37<br>[1.81, 3.43] | −3.30<br>[−7.41, −1.72] | 0.91<br>[0.62, 1.13] | 1.08<br>[0.67, 2.09] | 1.45<br>[1.23, 1.84] | 2.49<br>[1.98, 3.84] | 0.87<br>[0.79, 0.95] |
| missing-reject<br>& missing-reject-sampling | −0.15<br>[−0.32, −0.08] | 2.55<br>[1.80, 3.48] | −3.48<br>[−7.77, −1.92] | 0.85<br>[0.67, 1.15] | 1.19<br>[0.69, 2.19] | 1.41<br>[1.19, 1.81] | 3.78<br>[1.96, 8.22] | 0.81<br>[0.72, 0.92] |

bioRxiv preprint doi: https://doi.org/10.64898/2026.09.24.754193; this version posted September 29, 2026. The copyright holder for this preprint (which was not certified by peer review) is the author/funder, who has granted bioRxiv a license to display the preprint in perpetuity. It is made available under a CC-BY 4.0 International license.

301 ## 1.17 Spectral gap analysis on simulated dataset

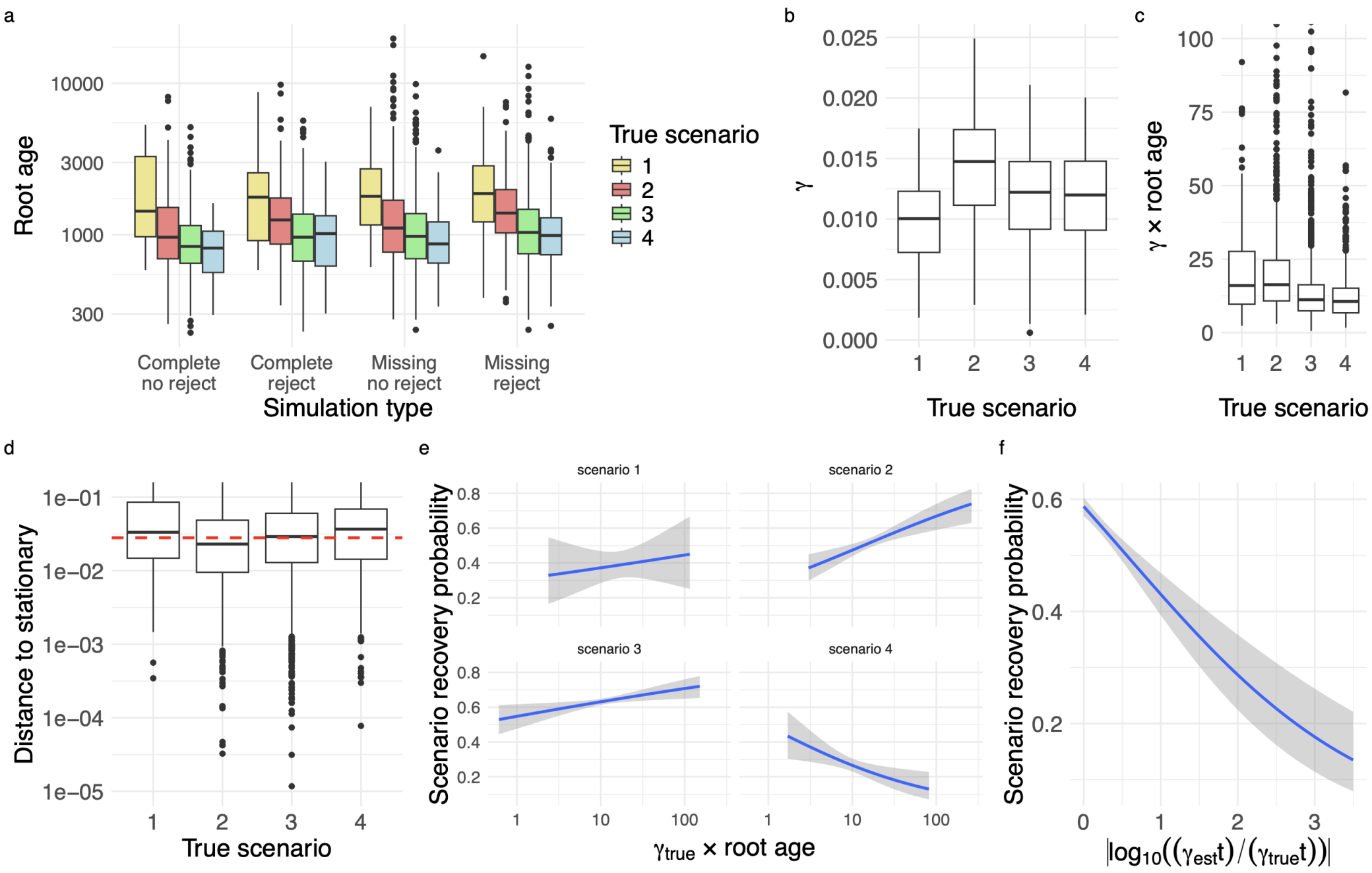


**Fig. S3**: Convergence properties and evolutionary-scenario recovery for datasets simulated using slow parameter rates. (a) Distribution of root ages across the four simulation settings, as described in Table 1, grouped by true evolutionary scenario. (b) Distribution of the spectral gap, $\gamma$, computed using the true generating parameters for each evolutionary scenario. (c) Distribution of the product $\gamma t$, where $t$ denotes the root age. (d) Distance between the observed tip state frequencies and the stationary frequencies under the true generating process for each evolutionary scenario. (e) Probability of correctly recovering the true evolutionary scenario as a function of $\gamma_{\text{true}} t$. Corresponding "a" and "b" directionality classes are pooled by scenario number for fitting and visualization, but recovery requires an exact match between the estimated and true evolutionary scenario, including the directionality class (e.g., 2a is correctly recovered only when inferred as 2a, not 2b). (f) Probability of correctly recovering the true evolutionary scenario as a function of the absolute log-ratio between the estimated and true spectral gaps, $|\log_{10}(\gamma_{\text{est}}/\gamma_{\text{true}})|$. Lines in panels (e) and (f) show fitted binomial logistic regressions, with shaded regions indicating 95% confidence intervals. For all boxplots, center black lines indicate medians, boxes span the interquartile range (IQR), whiskers extend to the most extreme observations within 1.5× the IQR, and points beyond the whiskers indicate outliers.

bioRxiv preprint doi: https://doi.org/10.64898/2026.09.24.754193; this version posted September 29, 2026. The copyright holder for this preprint (which was not certified by peer review) is the author/funder, who has granted bioRxiv a license to display the preprint in perpetuity. It is made available under a CC-BY 4.0 International license.

## 1.18 Confusion matrix using trees with missing taxa and without rejection on imbalanced tip states

**Table S4**: Prediction accuracy for each true scenario versus all other scenarios using the missing-noreject experiment. Scenarios 5a/b, 6a/b, 7a/b, 1c, 2c, and 3c are omitted because they do not have valid tree representation

| | | **Predicted scenario** | |
|---|---|---|---|
| | | **1a** | **Other scenarios** |
| True scenario | **1a** | 0.44 | 0.56 |
| | **Other scenarios** | 0.02 | 0.98 |
| | | **2a** | **Other scenarios** |
| | **2a** | 0.53 | 0.47 |
| | **Other scenarios** | 0.08 | 0.92 |
| | | **3a** | **Other scenarios** |
| | **3a** | 0.65 | 0.35 |
| | **Other scenarios** | 0.09 | 0.91 |
| | | **4a** | **Other scenarios** |
| | **4a** | 0.15 | 0.85 |
| | **Other scenarios** | 0.02 | 0.98 |
| | | **1b** | **Other scenarios** |
| | **1b** | 0.36 | 0.64 |
| | **Other scenarios** | 0.02 | 0.98 |
| | | **2b** | **Other scenarios** |
| | **2b** | 0.54 | 0.46 |
| | **Other scenarios** | 0.12 | 0.88 |
| | | **3b** | **Other scenarios** |
| | **3b** | 0.64 | 0.36 |
| | **Other scenarios** | 0.17 | 0.83 |
| | | **4b** | **Other scenarios** |
| | **4b** | 0.28 | 0.72 |
| | **Other scenarios** | 0.02 | 0.98 |

bioRxiv preprint doi: https://doi.org/10.64898/2026.09.24.754193; this version posted September 29, 2026. The copyright holder for this preprint (which was not certified by peer review) is the author/funder, who has granted bioRxiv a license to display the preprint in perpetuity. It is made available under a CC-BY 4.0 International license.

## 1.19 Confusion matrix using trees with missing taxa and without rejection on imbalanced tip states and account for sampling proportion

**Table S5**: Prediction accuracy for each true scenario versus all other scenarios using the missing-noreject-sampling experiment. Scenarios 5a/b, 6a/b, 7a/b, 1c, 2c, and 3c are omitted because they do not have valid tree representation.

| | | **Predicted scenario** | |
|---|---|---|---|
| | | **1a** | **Other scenarios** |
| **True scenario** | **1a** | 0.44 | 0.56 |
| | **Other scenarios** | 0.02 | 0.98 |
| | | **2a** | **Other scenarios** |
| | **2a** | 0.59 | 0.41 |
| | **Other scenarios** | 0.12 | 0.88 |
| | | **3a** | **Other scenarios** |
| | **3a** | 0.52 | 0.48 |
| | **Other scenarios** | 0.08 | 0.92 |
| | | **4a** | **Other scenarios** |
| | **4a** | 0.15 | 0.85 |
| | **Other scenarios** | 0.02 | 0.98 |
| | | **1b** | **Other scenarios** |
| | **1b** | 0.39 | 0.61 |
| | **Other scenarios** | 0.03 | 0.97 |
| | | **2b** | **Other scenarios** |
| | **2b** | 0.61 | 0.39 |
| | **Other scenarios** | 0.16 | 0.84 |
| | | **3b** | **Other scenarios** |
| | **3b** | 0.50 | 0.50 |
| | **Other scenarios** | 0.13 | 0.87 |
| | | **4b** | **Other scenarios** |
| | **4b** | 0.21 | 0.79 |
| | **Other scenarios** | 0.03 | 0.97 |

bioRxiv preprint doi: https://doi.org/10.64898/2026.09.24.754193; this version posted September 29, 2026. The copyright holder for this preprint (which was not certified by peer review) is the author/funder, who has granted bioRxiv a license to display the preprint in perpetuity. It is made available under a CC-BY 4.0 International license.

## 1.20 Confusion matrix using trees with complete sampling and with rejection on imbalanced tip states

**Table S6**: Prediction accuracy for each true scenario versus all other scenarios using the complete-reject experiment. Scenarios 5a/b, 6a/b, 7a/b, 1c, 2c, and 3c are omitted because they do not have valid tree representation.

| | | **Predicted scenario** | |
|---|---|---|---|
| | | **1a** | **Other scenarios** |
| **True scenario** | **1a** | 0.45 | 0.55 |
| | **Other scenarios** | 0.06 | 0.94 |
| | | **2a** | **Other scenarios** |
| | **2a** | 0.51 | 0.49 |
| | **Other scenarios** | 0.10 | 0.90 |
| | | **3a** | **Other scenarios** |
| | **3a** | 0.50 | 0.50 |
| | **Other scenarios** | 0.10 | 0.90 |
| | | **4a** | **Other scenarios** |
| | **4a** | 0.29 | 0.71 |
| | **Other scenarios** | 0.03 | 0.97 |
| | | **1b** | **Other scenarios** |
| | **1b** | 0.42 | 0.58 |
| | **Other scenarios** | 0.06 | 0.94 |
| | | **2b** | **Other scenarios** |
| | **2b** | 0.55 | 0.45 |
| | **Other scenarios** | 0.13 | 0.87 |
| | | **3b** | **Other scenarios** |
| | **3b** | 0.46 | 0.54 |
| | **Other scenarios** | 0.11 | 0.89 |
| | | **4b** | **Other scenarios** |
| | **4b** | 0.34 | 0.66 |
| | **Other scenarios** | 0.04 | 0.96 |

bioRxiv preprint doi: https://doi.org/10.64898/2026.09.24.754193; this version posted September 29, 2026. The copyright holder for this preprint (which was not certified by peer review) is the author/funder, who has granted bioRxiv a license to display the preprint in perpetuity. It is made available under a CC-BY 4.0 International license.

## 1.21 Confusion matrix using trees with missing taxa and with rejection on imbalanced tip states

**Table S7**: Prediction accuracy for each true scenario versus all other scenarios using the missing-reject experiment. Scenarios 5a/b, 6a/b, 7a/b, 1c, 2c, and 3c are omitted because they do not have valid tree representation.

| | | **Predicted scenario** | |
|---|---|---|---|
| | | **1a** | **Other scenarios** |
| **True scenario** | **1a** | 0.35 | 0.65 |
| | **Other scenarios** | 0.04 | 0.96 |
| | | **2a** | **Other scenarios** |
| | **2a** | 0.49 | 0.51 |
| | **Other scenarios** | 0.08 | 0.92 |
| | | **3a** | **Other scenarios** |
| | **3a** | 0.61 | 0.39 |
| | **Other scenarios** | 0.12 | 0.88 |
| | | **4a** | **Other scenarios** |
| | **4a** | 0.22 | 0.78 |
| | **Other scenarios** | 0.04 | 0.96 |
| | | **1b** | **Other scenarios** |
| | **1b** | 0.42 | 0.58 |
| | **Other scenarios** | 0.04 | 0.96 |
| | | **2b** | **Other scenarios** |
| | **2b** | 0.44 | 0.56 |
| | **Other scenarios** | 0.08 | 0.92 |
| | | **3b** | **Other scenarios** |
| | **3b** | 0.63 | 0.37 |
| | **Other scenarios** | 0.15 | 0.85 |
| | | **4b** | **Other scenarios** |
| | **4b** | 0.22 | 0.78 |
| | **Other scenarios** | 0.04 | 0.96 |

bioRxiv preprint doi: https://doi.org/10.64898/2026.09.24.754193; this version posted September 29, 2026. The copyright holder for this preprint (which was not certified by peer review) is the author/funder, who has granted bioRxiv a license to display the preprint in perpetuity. It is made available under a CC-BY 4.0 International license.

### 1.22 Confusion matrix using trees with missing taxa and with rejection on imbalanced tip states and account for sampling proportion

**Table S8**: Prediction accuracy for each true scenario versus all other scenarios using the missing-reject-sampling experiment. Scenarios 5a/b, 6a/b, 7a/b, 1c, 2c, and 3c are omitted because they do not have valid tree representation.

| | | Predicted scenario | |
|---|---|---|---|
| | | **1a** | **Other scenarios** |
| True scenario | **1a** | 0.41 | 0.59 |
| | **Other scenarios** | 0.05 | 0.95 |
| | | **2a** | **Other scenarios** |
| | **2a** | 0.55 | 0.45 |
| | **Other scenarios** | 0.11 | 0.89 |
| | | **3a** | **Other scenarios** |
| | **3a** | 0.52 | 0.48 |
| | **Other scenarios** | 0.10 | 0.90 |
| | | **4a** | **Other scenarios** |
| | **4a** | 0.22 | 0.78 |
| | **Other scenarios** | 0.04 | 0.96 |
| | | **1b** | **Other scenarios** |
| | **1b** | 0.49 | 0.51 |
| | **Other scenarios** | 0.06 | 0.94 |
| | | **2b** | **Other scenarios** |
| | **2b** | 0.54 | 0.46 |
| | **Other scenarios** | 0.12 | 0.88 |
| | | **3b** | **Other scenarios** |
| | **3b** | 0.52 | 0.48 |
| | **Other scenarios** | 0.12 | 0.88 |
| | | **4b** | **Other scenarios** |
| | **4b** | 0.19 | 0.81 |
| | **Other scenarios** | 0.04 | 0.96 |

## References

Goldberg EE, Lancaster LT, Ree RH (2011) Phylogenetic inference of reciprocal effects between geographic range evolution and diversification. Syst Biol 60(4):451–465

bioRxiv preprint doi: https://doi.org/10.64898/2026.09.24.754193; this version posted September 29, 2026. The copyright holder for this preprint (which was not certified by peer review) is the author/funder, who has granted bioRxiv a license to display the preprint in perpetuity. It is made available under a CC-BY 4.0 International license.

Soewongsono AC, Landis MJ (2024) A diffusion-based approach for simulating forward-in-time state-dependent speciation and extinction dynamics. arXiv preprint arXiv:240200246

Wolfram Research, Inc. (2023) Mathematica. URL https://www.wolfram.com/mathematica
